# Self-concept clarity and Internet addiction disorder among junior high school students: A moderate mediation model

**DOI:** 10.3389/fpsyt.2022.989128

**Published:** 2022-08-18

**Authors:** Yue Wang, Weiyi Tang, Lei Cao, Ying Li

**Affiliations:** School of Education, Zhengzhou University, Zhengzhou, China

**Keywords:** junior high school students, Internet addiction disorder, self-concept clarity, cognitive failure, mindfulness

## Abstract

**Introduction:**

As the epidemic spreads, the problem of Internet addiction disorder (IAD) stand out and getting serious. The present study aimed to investigate IAD among junior high school students during the spread of the COVID-19, and to explore the mediating role of cognitive failure between self-concept clarity and IAD, and the moderating role of mindfulness.

**Methods:**

A sample of 1,153 junior high school students from two randomly selected junior high schools in Henan Province were surveyed anonymously with Self-concept Clarity Scale (SCCS), Cognitive Failure Questionnaire (CFQ), Mindfulness Attention Awareness Scale (MAAS) and Internet Addiction disorder Test (IAT). The sample was obtained through random cluster sampling, taking classes as the clusters and students as the elements.

**Results:**

(1) Self-concept clarity was negatively correlated with Internet addiction disorder; (2) Self-concept clarity not only had a direct effect on Internet addiction disorder, but also indirectly affect Internet addiction disorder through cognitive failure; (3) Mindfulness moderates the relationship between self-concept clarity and Internet addiction disorder, as well as the relationship between cognitive failure and Internet addiction disorder. Compared with low levels of mindfulness, both the protective effect of self-concept clarity and the effect of cognitive failure on Internet addiction disorder were stronger among junior high school students who were at high levels of mindfulness.

**Conclusion:**

This study constructs a moderated mediation model to explain the effect of self-concept clarity on Internet addiction disorder. It is effective to alleviate Internet addiction disorder by improving self-concept clarity and mindfulness level of the junior school students.

## Introduction

The Internet has played an important role in people's lives and work, especially during the COVID-19 pandemic. The Coronavirus disease 2019 (COVID-19) as a public health emergency brought about a sharp increase in the number of behavioral addictions (1). Behavioral addictions, especially Internet addiction disorder, have become more prominent among adolescents during the COVID-19 pandemic (2). In order to deal with the spread of the virus, various countries and regions have launched different strategies, such as reducing aggregation, locking down cities, shutdown or home office, school suspension or e-learning. Although these measures are meant to protect people from the virus, they also bring new problems, especially Internet addiction disorder among teenagers. During the pandemic, they need to temporarily stop the traditional face-to-face learning and rely more on online distance learning. Since online learning is generally unsupervised, it is easy for teenagers to indulge in Internet surfing or games. Moreover, adolescents' self-awareness and personality have not yet developed well, and it has not been fully developed in their self-control ability. Coupled with the attraction of the Internet world, teenagers are always a high incidence group of Internet addiction disorder (3). At the same time, due to the fear of viruses and the depression and stress caused by uncertainty, junior high school students tend to seek sense of security from cyberspace (4). With the increasing number of minors using the network, Low age in the problem of Internet addiction disorder gets worse with each passing day (5). Previous researches provided a global internet addiction disorder prevalence of 7.02%, and the prevalence was 7.7% in Chinese middle school students (6).

Internet addiction disorder (IAD) is defined as the loss of control over Internet use (7, 8) leads to a series of physical and mental health and social adaptation problems in everyday life, such as decreased sleep quality, decreased academic performance, interpersonal tension and many other negative effects (9). In view of the negative effects of IAD, it is particularly urgent to solve the problem of IAD among teenagers. The society is paying much attention to the IAD of college students, many researches were done from different kinds of fields. In this context, it is necessary to explore the influencing factors and psychological mechanism of middle school students' IAD, which has practical significance to reduce and solve the problem of IAD.

Self-concept clarity is a structured self-concept, which refers to an individual's confidence and clarity in self-evaluation and self-knowledge (10). The influencing factors of IAD is still a topic ongoing and under investigation, and self-concept clarity is one of the most important factors. Previous studies with adult subjects have found that self-concept clarity is significantly and negatively associated with IAD. Specifically speaking, low level of self-concept clarity may result in Internet-dependent behaviors and eventually it becomes an addiction (11, 12). The reason is that individuals with low self-concept clarity usually show more inferiority and introversion, pay too much attention to the inner world, and do not have a comprehensive understanding of their own abilities. Therefore, they are more likely to indulge in alternative satisfaction through the Internet (13). It was found that the level of self-concept clarity of middle school students was slightly lower than the theoretical mean level (14). Some studies have found that adolescents with a low level of self-concept clarity typically have more negative coping styles, poorer interpersonal relationships, and exhibit more anxiety and depression (15). Evidence from relevant reviews have suggested that there is a significant correlation between self-concept clarity and IAD, but few studies have examined whether this relationship is the same for junior middle school students. Adolescents are more curious but lack mature cognitive control, self-regulatory, and more likely to suffer from environmental adjustment, and other life events. Based on this, this paper will take junior high school students as the research object to explore the impact of their self-concept clarity on IAD.

### Mediating role of cognitive failures

Cognitive failure refers to perceptual, memory or motor lapses that occur in daily life, as well as multiple aspects of subjective cognitive failures—including decision-making, attention, and memory (16). Generally speaking, individuals with low self-concept clarity tend to experience more negative emotions and lower self-evaluation (17). According to Conservation of Resources Theory, individuals with low self-evaluations are inclined to accumulate negative emotions and thus consume more cognitive resources. Excessive self-concern and negative emotions also make it difficult for individuals to allocate cognitive resources effectively when dealing with daily life events, which leads to cognitive failure (18). The results of several empirical studies confirm the relationship between self-concept clarity and cognitive failure (19). For example, social anxiety is alleviated as adolescents' self-concept clarity improves. Hence, low self-concept clarity is significant factor in causing individual social anxiety (20), while social anxiety is widely recognized as one of the important causes of cognitive failure (21). Specifically, compared with high self-concept clarity, individuals with low self-concept clarity are unable to correctly deal with the negative effects of social anxiety and are prone to self-attrition, which caused ultimately cognitive failure (22). In addition, there is a distinct link between cognitive failure and IAD. Cognitive failure contribute to the reduction of cognitive resources, which results in competition between the needs of daily activities and the excessive use of the Internet, and IAD is easy to occur under competitive conditions (23). Unsworth et al. noted that individuals with cognitive failure have less working memory capacity and poorer cognitive control, which makes them more susceptible to the temptation of the Internet (24). Since individuals can compensate for their failure behaviors in daily life through the Internet (25), which will further promote the frequency and time of using the Internet and increase the likelihood of IAD.

### Moderating role of mindfulness

However, not all the individuals with low self-concept clarity and high cognitive failure necessarily lead to serious psychological or behavioral problems. It has found that there are individual differences in the negative effects of self-concept clarity and cognitive failure on adolescents, that is, such effects may be moderated by individual characteristics and then affect individual behavior (26). Mindfulness as an individual trait refers to an open, accepting attitude in which individuals purposefully and non-judgmentally direct their attention to current experiences (27–29). Mindfulness may reduce the negative effects of cognitive failure (30). According to the two-factor model of mindfulness, mindfulness emphasizes the individual's ability to pay attention to current experiences non-judgmentally. It can help individuals perceive things more comprehensively and objectively (31). Moreover, A large number of studies have found that mindfulness has a relatively positive impact on individual self-development, physical health, cognitive, emotional and behavioral problems (30, 32), also confirmed the protective effect of mindfulness on addictive behaviors (33–36). Mindfulness may provide individuals the ability to self-regulate their emotional and reduce their responses to potentially emotional and stressful stimuli by improving awareness (37). For example, mindfulness intervention for college students can effectively reduce their mobile phone dependence (38). However, existing results are still controversial, with some studies suggesting that mindfulness does not always have “ideal” effects (39, 40). Dubow et al. examined the contribution of particular stressors and resources to children's adjustment and found that peer support not only did not alleviate but exacerbated the effect of stressors on behavioral maladjustment (41). Although mindfulness can positively influence individual behavior, its protective effect has some limitations. In other words, while positive traits such as mindfulness are protective, it is not known whether they can protect individuals against the negative effects of risk factors such as cognitive failure. The effect of mindfulness on self-concept definition and IAD needs further research.

### The present study

**Figure 1:**
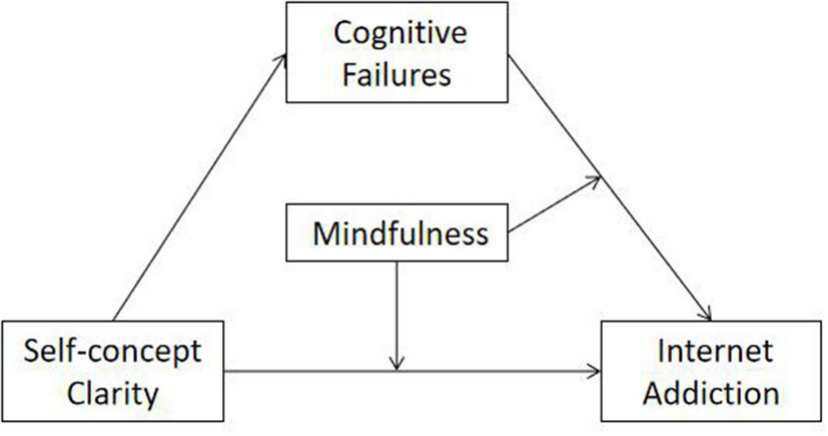
Proposed research model.

In this study, we aimed to explore the association between self-concept clarity and IAD. Therefore, we constructed a moderated mediation model to test the mediating effect of cognitive failure on self-concept clarity and IAD, as well as the moderating effect of mindfulness (see Figure 1). Specifically, we proposed the following hypotheses:

**Hypothesis 1 (H1):** Self-concept clarity is negatively correlated with IAD.
**Hypothesis 2 (H2):** Cognitive failures mediates the relationship between self-concept clarity and IAD.
**Hypothesis 3 (H3):** Mindfulness moderates the relationship between self-concept clarity and IAD, as well as the relationship between cognitive failure and IAD.

## Methods

### Participants and procedure

The participants are students from three grades in two randomly selected junior high schools in Henan Province. The sample was obtained through random cluster sampling, taking classes as the clusters and students as the elements. A total of 1,400 questionnaires were distributed, and the data from 1,153 (82.36%) participants were collected. Of these participants, 610 (52.9%) participants were female and 543 (47.1%) were male, 387 of them are first-year students, 274 of them are second-year students, 492 of them are third-year students. Participants' age ranged from 11 to 17 years, with a mean age of 13.84 ± 2.82 years. Informed consent was obtained from all participants and no compensation was provided to participants. Comparing the structures of the population and he sample, the main relevant indicators of the sample were consistent with the overall structure of the junior high schools population, indicating good sample representativeness.

The study was conducted in a classroom setting, and the entire process was conducted by a trained psychology student who used uniform instructions prior to administration, and the questionnaire was completed anonymously and collected on the spot upon completion.

### Measures

#### Self-concept clarity

We used Campbell et al.'s Self-Concept Clarity Scale (10). This is a 12-item scale on the 5-point Likert scale ranging from 1 = strongly disagree to 5 = strongly agree. A sample item is “My beliefs about myself often conflict with one another.” In this study, the internal consistency of the SCCS was 0.68.

#### Cognitive failures

The CFQ consists of 25 items assessing five dimensions of cognitive failures: distractibility, memory, interpersonal, blunders, motor coordination, and memory for names. Items are scores on a 5-point Likert scale ranging from 1 = never, to 5 = always. Higher scores indicate increasing

behavioral errors caused by cognitive distortions. Sample items include: “I found myself suddenly wondering if I had just worded it correctly”; “I don't know what I want to say”; and “Can't tell left from right when giving directions.” In the present study, the Cronbach's α coefficient of this scale was 0.92.

#### Mindfulness attention awareness scale

The MAAS is a 15-item measure of mindfulness. Items are rated on a 6-point Likert scale ranging from 1 = almost always, to 6 = almost never. Sample items include: “I can't focus very well on what's happening at the moment”; “I always complete a task mechanically and without consciousness”; and “I would focus too much on the future or the past”. Higher scores represent higher levels of mindfulness. In our study, the Cronbach's α coefficient of scores from this scale was 0.86.

#### Internet addiction disorder

In order to measure IAD, we used Young's (7) Internet Addiction disorder Questionnaire. This questionnaire has been widely used in various studies and has already been translated to other languages, such as Chinese, Norwegian and Italian. Respondents are asked to rank their responses on a 4-point Likert scale, ranging from 1, “completely untrue,” to 4, “completely true.” In the current study, the Cronbach alpha for the entire scale was 0.87.

### Data analysis

All data analyses were conducted using SPSS 21.0. First, we computed descriptive statistics and performed correlation analysis of the variables. Second, a simple mediation analysis of cognitive failures mediating the relationship between role self-concept clarity and IAD was tested using Hayes's PROCESS macro for SPSS(Model 4). Third, a moderated mediation analysis was conducted using Hayes' PROCESS macro (Model 15) to test the moderating role of mindfulness in direct and indirect effects of cognitive failures on IAD in the mediation model. The indirect effect of mediation was tested using a bootstrapping method with 5,000 samples as recommended, with a significant effect indicated by a 95% confidence interval not including zero.

## Results

### Common-method variance test

An explanatory factor analysis (EFA) including all variables using unrotated principal components factor analysis was performed to statistically verify the presence of CMV. The results revealed that 13 factors had eigenvalues >1, and the general factor accounted for only 23.48% of the total variance, which did not exceed the critical value of 40%.It concluded that CMV was not a concern.

### Descriptive statistics and correlation analysis

Table 1 lists the means and standard deviation of the study variables and their correlations. The results indicate that IAD had significant positive correlations with cognitive failures, and had significant negative correlation with self-concept clarity and mindfulness. Cognitive failures had significant negative correlation with self-concept clarity. Besides, age and gender were significantly correlated with some variable. Therefore, both were treated as control variables in the subsequent analysis.

**Table 1:** Descriptive statistics and correlations among variables.

		**1**	**2**	**3**	**4**	**5**	**6**
1 Age	12.72 ± 2.82	1	-	-	-	-	-
2 Gender	1.53 ± 0.50	−0.04	1	-	-	-	-
3 Self-concept clarity	34.05 ± 5.94	0.11^**^	−0.01	1	-	-	-
4 Cognitive failures	69.17 ± 16.41	−0.05	0.11^**^	−0.25^**^	1	-	-
5 Mindfulness	53.50 ± 12.50	0.04	−0.01	0.28^**^	−0.64^**^	1	-
6 Internet addiction	18.17 ± 6.87	−0.11^**^	−0.12^**^	−0.28^**^	0.47^**^	−0.43^**^	1

** *p < 0.01, two-tailed test*.

### Testing for mediating effect

Table 2 shows the results of mediating effect of cognitive failures. Self-concept clarity had a significant negative effect on IAD (β = −0.26, t = −9.52, *p* < 0.001), and on cognitive failures (β = −0.24, t = −8.60, *p* < 0.001). When the mediating variable cognitive failures was added, the direct effect of self-concept clarity on IAD was still significant (β = −0.16, *t* = −6.68, *p* < 0.001), indicating partial meditation. Cognitive failures had a significant positive effect on IAD (β = 0.44, t = 16.87, *p* < 0.001).

**Table 2:** Testing the mediation effect of self-concept clarity on IAD through cognitive failures.

	**Model 1(IAD)**		**Model 2 (Cognitive failures)**		**Model 3 (IAD)**	
	**β**	**t**	**β**	**t**	**β**	**t**
Age	−0.03	−3.00**	−0.01	−0.76	−0.03	−2.97**
Gender	−0.24	−4.36^***^	0.21	3.72^***^	−0.33	−6.68^***^
Self-concept clarity	−0.26	−9.52^***^	−0.24	−8.60^***^	−0.16	−6.15^***^
Cognitive failures					0.44	16.87^***^
R^2^	0.1		0.07		0.28	
F	41.31^***^		30.48^***^		109.76^***^	
P	0.001		0.001		0.001	

*Each column is a regression on model that predicts the criterion at the top of the column*.

*** *P < 0.001*.

Further bootstrapping results showed cognitive failures partially mediated the relationship between self-concept clarity and IAD, with an indirect effect accounting for 40.28% of the total effect.

### Testing for moderated mediation

In our proposed moderation mediation model, we hypothesized that mindfulness would moderate direct and indirect effects of cognitive failures on IAD in the mediation model. Table 3 shows the results of such moderation mediation analysis using Model 15 of PROCESS macro by Hayes. After mindfulness was put into the model, self-concept clarity had a significant negative effect on cognitive failures (β = −0.24, *t* = −8.60, *p* < 0.001), cognitive failures had significant positive effect on IAD (β = 0.34, *t* = 10.41, *p* < 0.001), and self-concept clarity had a significant negative effect on cognitive failures (β = −0.14, *t* = −5.31, *p* < 0.001. In addition, the product of self-concept clarity and mindfulness had a significant positive effect on IAD (β = 0.06, *t* = 2.71, *p* < 0.01), and the product of cognitive failures and mindfulness had a significant positive effect on IAD (β = 0.06, *t* = 2.71, *p* < 0.01), indicating that social support moderated the direct effect of self-concept clarity and indirect effects of cognitive failures.

**Table 3:** Testing the moderated mediation effect of mindfulness on the relation between self-concept clarity and IAD *via* cognitive failures.

	**Model 1**		**Model 2 (IAD)**	
	**(Cognitive failures)**			
	**β**	**t**	**β**	**t**
Age	−0.01	−0.76	−0.03	−2.90**
Gender	0.21	3.72^***^	−0.30	−6.15^***^
Self-concept clarity	−0.24	−8.60^***^	−0.14	−5.31^***^
Cognitive failures			0.34	10.41^***^
Mindfulness			−0.17	−5.14^***^
Self-concept clarity*Mindfulness			0.06	2.71**
Cognitive failures*Mindfulness			0.06	2.96**
R^2^	0.07		0.30	
F	30.48^***^		71.16^***^	
P	0.001		0.001	

*Each column is a regression model that predicts the criterion at the top of the column*.

*** *p < 0.001*.

**Figure 2:**
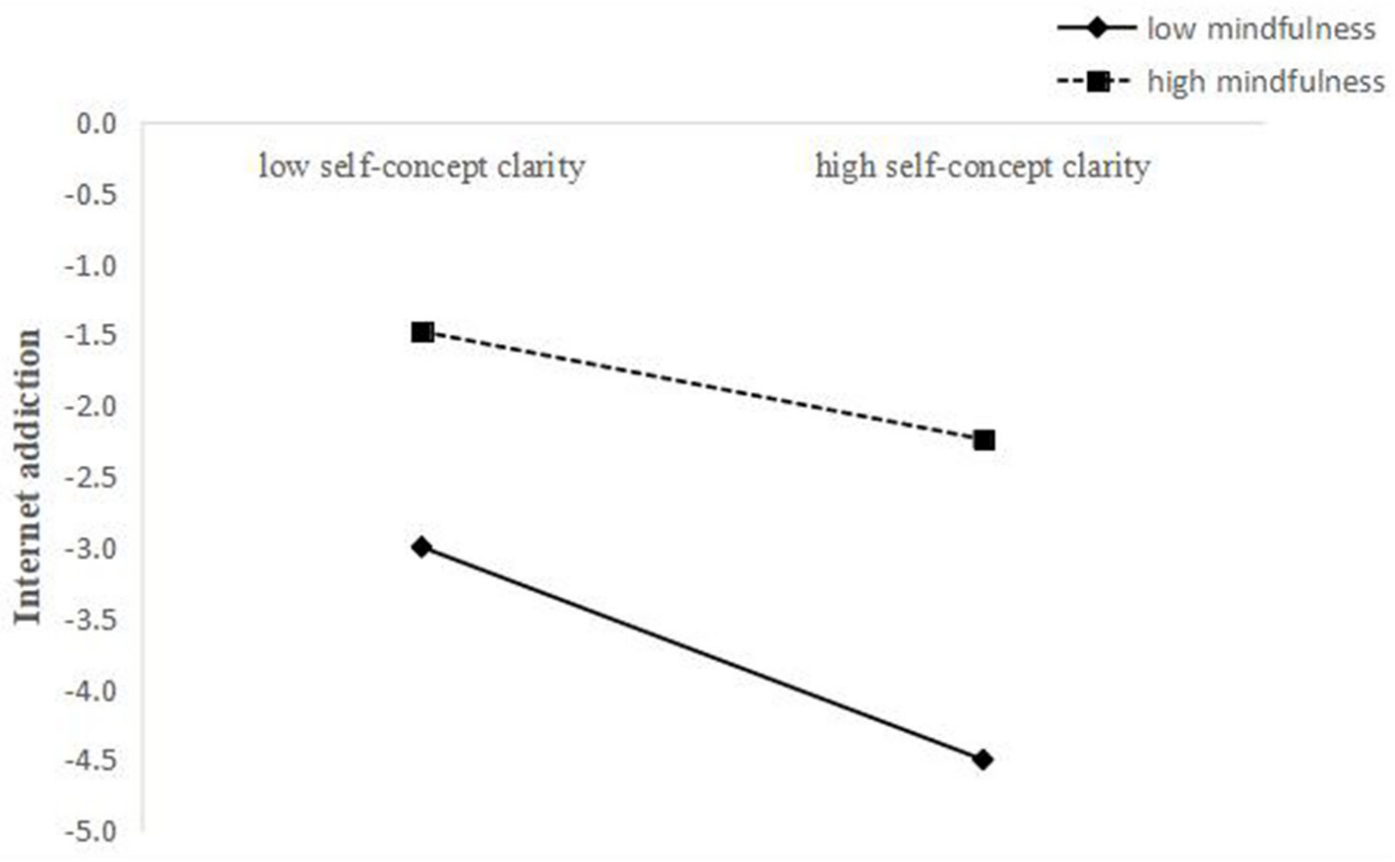
The interaction between self-concept clarity and mindfulness on IAD.

**Figure 3:**
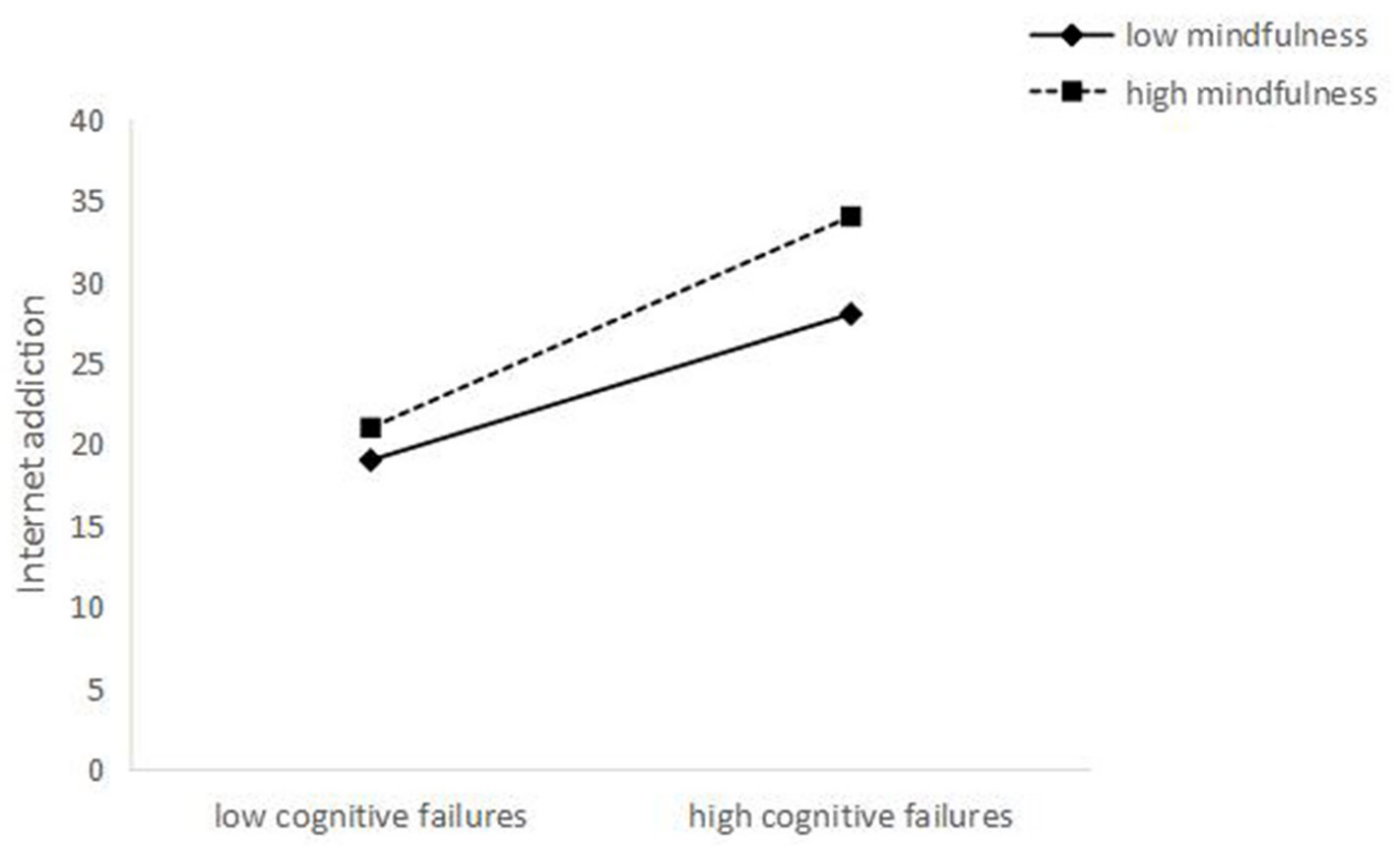
The interaction between cognitive failures and mindfulness on IAD.

To better interpret the moderating effects of mindfulness, we examined the simple effects of both cognitive failures on IAD and self-concept clarity on IAD, at different levels of mindfulness (1 SD below the mean and 1 SD above the mean). Simple slope tests showed that the association between self-concept clarity and IAD was stronger for individuals with low mindfulness (β = −0.20, t = −5.71, *p* < 0.001) than for individuals with high mindfulness (β = −0.08, t = −2.25, *p* < 0.05) (see Figure 2). Similarly, the association between cognitive failures and IAD was stronger for individuals with high mindfulness (β = 0.40, *t* = 9.81, *p* < 0.001) than for individuals with low mindfulness (β = 0.28, *t* = 7.54, *p* < 0.001) (see Figure 3).

## Discussion

This study explored the effect of self-concept clarity on IAD among junior middle school students during THE COVID-19 pandemic in China. The mechanism for this effects is briefly discussed. We found that self-concept clarity negatively predicted IAD, confirming the mediating role of cognitive failure and the moderating role of mindfulness.

### Self-concept clarity predicted IAD

Consistent with hypothesis 1, the lower the self-concept of junior middle school students, the more serious the IAD behavior. In other words, Self-concept clarity can significantly negatively predict IAD of junior high school students, which is consistent with previous research conclusions (11, 42). According to uncertainty-identity theory, uncertainty about the self is aversive and uncomfortable, and that uncertainty can cause negative emotional and cognitive responses, so people always try to reduce, control, or avoid the occurrence of self-uncertainty (43). Previous research has pointed out that higher self-concept clarity is an important indicator of adolescents' social adjustment, and adolescents with higher self-concept clarity also generally have higher levels of wellbeing and lower levels of depression, anxiety, and loneliness (44). The presence of these negative Emotion make adolescents with lower self-concept clarity tend to find “safe havens” in the Internet. According to the compensatory Internet use hypothesis, it is known that the network allows them to experience a sense of achievement or a sense of identity (25). Previous research has shown that instant gratification are important to individuals with low self-concept clarity and they are largely unwilling to delay gratification (45). In order to compensate for real life problems, junior high school students with low self-concept clarity tend to seek more internet resources such as online information, games or socialization (13, 25, 46). Couple with their own immature thinking and poor self-control, they have difficulty in resisting the temptation of the Internet. Compared to normal times, adolescents who use the Internet for long periods of time during the pandemic are more likely to develop addictive behaviors than in normal times (11). The results provide a new perspective to explain the Internet addictive behaviors of junior high school students in special periods, reveal the underlying causes of Internet addictive behaviors, and suggest that further attention should be paid to the negative effects of the lack of self-concept clarity on junior high school students.

### The mediating role of cognitive failures

As expected, cognitive failures partially mediated the relationship between self-concept clarity and IAD, supporting our hypotheses 2. Junior high school students with lower self-concept clarity tend to be more prone to cognitive failure, which leads to IAD.

For the first path of the mediation process, we found that self-concept clarity was negatively linked to cognitive failure. On the one hand, if junior high school students have a clear understanding of self-concept, they will have higher self-evaluation and more positive emotional experience, which will help them allocate cognitive resources more rationally and effectively in the face of daily life events (46) and reduce cognitive failure in daily life. Conversely, negative self-evaluation enhances the activation of idiosyncratic failure episodes, leading to the preferential retrieval of this part of the individual's memory events. Therefore, individuals with lower self-concept clarity are less likely to take effective measures to cope with events, and ultimately leading to cognitive failure (18, 47). On the other hand, studies have shown that individuals' self-concept clarity is significantly negatively correlated with neuroticism (N) (48), while individuals with high neuroticism are more prone to cognitive failure, and they are more likely to ignore task-related information and be easily disturbed by task-unrelated information (49). According to the mental noise hypothesis of neuroticism, individuals with high neuroticism are more susceptible to psychological noise, which affects executive function and ultimately result in cognitive failure, than individuals with low neuroticism (50).

For the second path of the mediation process, this study indicated that cognitive failure was positively related to IAD, which supported the previous researches (51). The results, to a certain extent, validate the hypothesis of compensatory Internet use. According to this hypothesis, the junior high school students with high level of cognitive failure did not take the right way to cope with the failures they experienced in daily life in time, but re-experienced “compensatory success” through the pathological compensation of frequent Internet use (52). This undesirable cycle will eventually lead to IAD (25). Individuals with high cognitive failure are characterized by inefficiency of executive functioning and high trait impulsivity (53), which makes them more likely perform high-risk behaviors such as pathological gambling and risky sexual behavior (54). Besides, substance-related addictions is more severe and persistent in individuals with high cognitive failure (55). And there are many similarities between behavioral additions and substance-related addictions such as comorbidity, genetics, and neurobiological mechanisms (56). Studies have shown that individuals with high levels of attentional inattention and attentional lapses in daily life generalize this tendency to cell phone use, producing problematic cell phone use behaviors, such as using their phones for longer than they expect, continuously browsing for information without purposes, and repeatedly checking their phones (57). It not only supports the compensatory Internet use hypothesis of Conservation of Resources Theory, but also confirms the mediating role of cognitive failure between self-concept clarity and IAD.

### The moderating role of mindfulness

A higher level of perceived stress of the COVID-19 pandemic might lead to a sharp increase in the number of mental health problem, such as post-traumatic stress symptoms, confusion and depression (58). At the same time, previous researches suggested that mindfulness training can play a significant role in relieving pain, improving cognition and regulating emotions by improving people's level of mindfulness (59, 60). It will help junior high school students respond to the outside world in a more flexible way, thus avoiding Internet addiction (61). Our study further found mindfulness moderated the indirect association between self-concept clarity and IAD among junior high school students through cognitive failure during the COVID-19 pandemic, supporting our hypotheses 3. To be specific, in terms of the direct effect, the protective effect of high self-concept clarity on IAD increased with the level of mindfulness, which is consistent with most previous studies (62, 63). Junior high school students with high levels of mindfulness are able to confront their own characteristics, and they are not obsessed by their deficiencies and focuses on current experiences. Therefore, junior high school students with high level of mindfulness can concentrate on their studies on their studies without interference, thus avoiding IAD (64). According to the mindfulness reperceiving model, “perception” helps individuals to accept life events in a more objective way (65). Mindfulness is a significant protective factor of self-efficacy and sense of security (66), which will help students cope with the various stressors around them. If students are able to cope with stress effectively, the negative effects of IAD will be gradually mitigated accordingly.

The result also suggested Mindfulness moderated the relationships between cognitive failures and IAD. To be specific, compared with low levels of mindfulness, the effect of cognitive failure on IAD was stronger among junior high school students who were at high levels of mindfulness. There may be two reasons for this: First, the impact of cognitive failure is far-reaching. Junior high school students with high cognitive failure hardly experience “success” in their daily lives, and the “compensatory success” given by the online world makes them feel they can handle their own lives. Second, Individuals with cognitive failure generally have fewer cognitive resources, and mindfulness requires individuals to detach from their experience and accept the event objectively, which still takes up a lot of cognitive resources of junior high school students. In this way, the two processes compete for cognitive resources which increase the likelihood of cognitive failure (67, 68). While the results of this study not only provide some evidence that mindfulness is a protective factor for IAD, they also suggest that risk factors may undermine the positive effects of protective factors in the association between different factors (69). This result suggests that the interaction between mindfulness and other individual and social contextual factors should be taken into account when exploring the influence of mindfulness on IAD, so as to analyze the positive effects of mindfulness more comprehensively and objectively.

### Implications

The findings of this study provide a new perspective for the prevention, intervention and treatment of junior high school student's IAD. Firstly, consistent with other research, our findings suggest that self-concept clarity is a significant factor for IAD. This result indicates that intervention targeting avoiding IAD should start with improving students' self-concept clarity, which may be realized through the discussion of self-concept. For example, teachers can set up an interactive platform for students to freely talk about the hot issues in the society and guide them to learn more about themselves. Schools can provide group psychotherapy to enhance students' sense of self-awareness. Secondly, given that cognitive failure is a significant mediator linking self-concept clarity and IAD, avoiding cognitive failure may be another helpful and efficient measure to avoid IAD. For instance, psycho-social interventions aimed at focusing limited cognitive resources on academic activities and preventing intrusions of task-irrelevant information may best serve the purpose of avoiding IAD. Finally, although the protective effect of mindfulness has some limitations, it can positively influence individual behavior. Improving the level of mindfulness may also greatly help junior high school students avoid IAD. Specifically, school activities can be implemented and improved to enhance junior high school students' mindfulness level. For example, schools can provide group psychotherapy for students to increase mindfulness levels in adolescents, improve their cognitive style. Teachers can also offer psychology classes for students to teacher them interventions such as mindfulness-based and cognitive behavioral training. Because the Coronavirus is strongly contagious, compared with face-to-face posttraumatic stress disorder (PTSD) treatment, Internet-delivered interventions have more advantages in the epidemic (70, 71). As an important part of Internet-delivered interventions, online mindfulness-based interventions could help people concern in the process of inner strengths and abilities and establish close relationships with others (72), which will help junior high school students avoid Internet addiction.

### Limitations and future research directions

Although the present study advances our understanding of the mediating and moderating mechanism underlying the association between self-concept clarity and IAD among junior high school students, several limitations need to be acknowledged. First, this survey used a cross-sectional design, which leads to the inability to infer causality. Longitudinal studies could be carried out in the later study to further verify the moderated mediation model. Second, this study only measured individual variables of junior high school students; future studies could take into account environmental factors of junior high school students, such as parenting style and school climate. Third, we over-relied on self-report data, which is susceptible to social desirability bias. Future studies should collect information from multiple informants.

## Conclusion

Although some studies attention has been directed toward understanding the effect of self-concept clarity on IAD, less attention has been paid to the mediation and moderation mechanisms underlying such an association among junior high school students. This study suggests that self-concept clarity was associated with IAD both directly and indirectly through cognitive failure, both were further moderated by mindfulness. A high level of mindfulness may increase the protective effect of high self-concept clarity on IAD and the negative predictive effect of cognitive failure on IAD.

## Data availability statement

The raw data supporting the conclusions of this article will be made available by the authors, without undue reservation.

## Ethics statement

Ethical review and approval was not required for the study on human participants in accordance with the local legislation and institutional requirements. Written informed consent from the participants' legal guardian/next of kin was not required to participate in this study in accordance with the national legislation and the institutional requirements.

## Author contributions

YL and YW contributed to conception and design of the study. LC organized the database. WT performed the statistical analysis. YL and LC wrote the first draft of the manuscript. YW and WT wrote sections of the manuscript, contributed to manuscript revision, read, and approved the submitted version.

## Funding

This study was supported by the Educational Science Planning Project of Henan Province (Grants No. 2022JKZD32) and project of Humanities and Social Sciences from Ministry of Education in China, 20YJC190023.

## Conflict of interest

The authors declare that the research was conducted in the absence of any commercial or financial relationships that could be construed as a potential conflict of interest.

## Publisher's note

All claims expressed in this article are solely those of the authors and do not necessarily represent those of their affiliated organizations, or those of the publisher, the editors and the reviewers. Any product that may be evaluated in this article, or claim that may be made by its manufacturer, is not guaranteed or endorsed by the publisher.
